# Identification of factors associated with stillbirth in the Indian state of Bihar using verbal autopsy: A population-based study

**DOI:** 10.1371/journal.pmed.1002363

**Published:** 2017-08-01

**Authors:** Rakhi Dandona, G. Anil Kumar, Amit Kumar, Priyanka Singh, Sibin George, Mohammad Akbar, Lalit Dandona

**Affiliations:** 1 Public Health Foundation of India, Gurgaon, National Capital Region, India; 2 Institute for Health Metrics and Evaluation, University of Washington, Seattle, Washington, United States of America; The Hospital for Sick Children, CANADA

## Abstract

**Background:**

India was estimated to have the largest numbers of stillbirths globally in 2015, and the Indian government has adopted a target of <10 stillbirths per 1,000 births by 2030 through the India Newborn Action Plan (INAP). The objective of this study was to use verbal autopsy interviews to examine factors associated with stillbirth in the Indian state of Bihar and make recommendations for the INAP to better inform the setting of priorities and actions to reduce stillbirths.

**Methods and findings:**

Verbal autopsy interviews were conducted for deaths including stillbirths that occurred from January 2011 to March 2014 in a sample of 109,689 households (87.1% participation) in 1,017 clusters representative of the state of Bihar. The Population Health Metrics Research Consortium shortened verbal autopsy questionnaire was used for each interview, and cause of death was assigned using the SmartVA automated algorithm. A stillbirth was defined as a foetal death with a gestation period of ≥28 weeks wherein the foetus did not show any sign of life. We report on the stillbirth epidemiology and present case studies from the qualitative data on the health provider interface that can be used to improve success of improved, skilled care at birth and delivery interventions. The annualised stillbirth incidence was 21.2 (95% CI 19.7 to 22.6) per 1,000 births, with it being higher in the rural areas. A total of 1,132 stillbirths were identified; 686 (62.2%) were boys, 327 (29.7%) were firstborn, and 760 (68.9%) were delivered at a health facility. Of all the stillbirths, 54.5% were estimated to be antepartum. Only 6,161 (55.9%) of the women reported at least 1 antenatal care visit, and 33% of the women reported not consuming the iron folic acid tablets during pregnancy. Significant differences were seen in delivery-related variables and associated maternal conditions based on the place of delivery and type of stillbirth. Only 6.1% of the women reported having undergone a test to rule out syphilis. For 34.2% of the stillbirths, the possible risk factor for stillbirth was unexplained. For the remaining 65.8% of the women who reported at least 1 complication during the last 3 months of pregnancy, maternal conditions including anaemia, fever during labour, and hypertension accounted for most of the complications. Of importance to note is that the maternal conditions overlapped quite significantly with the other possible underlying risk factors for stillbirth. Obstetrics complications and excessive bleeding during delivery contributed to nearly 30% of the cases as a possible risk factor for stillbirth, highlighting the need for better skilled care during delivery. Of the 5 major themes identified in open narratives, 3 were related to healthcare providers—lack of timely attention, poor skills (knowledge or implementation), and reluctance to deliver a dead baby. The case studies document the circumstances that highlight breakdowns in clinical care around the delivery or missed opportunities that can be used for improving the provision of quality skilled care. The main limitation of these data is that stillbirth is defined based on the gestation period and not based on birth weight; however, this is done in several studies from developing country settings in which birthweight is either not available or accurate.

**Conclusions:**

To our knowledge, this study is among the few large, population-based assessments of stillbirths using verbal autopsy at the state level in India. These findings provide detailed insight into investigating the possible risk factors for stillbirths, as well as insight into the ground-level changes that are needed within the health system to design and implement effective preventive and intervention policies to reduce the burden of stillbirths. As most of the stillbirths are preventable with high-quality, evidence-based interventions delivered before and during pregnancy and during labour and childbirth, it is imperative that with INAP in place, India aspires to document stillbirths in a systematic and standardised manner to bridge the knowledge gap for appropriate actions to reduce stillbirths. We have made several recommendations based on our study that could further strengthen the INAP approach to improve the quality and quantity of stillbirth data to avoid this needless loss of lives.

## Abstract 2

In a verbal autopsy study, Rakhi Dandona and colleagues describe factors associated with stillbirth in the Indian state of Bihar.

## Author summary

**Why was this study done?:**

- An estimated 2.6 million third-trimester stillbirths occurred in 2015 globally, and India accounted for the largest numbers of these stillbirths.
- The first ever national stillbirth prevention target of <10 stillbirths per 1,000 births by 2030 was adopted by the Indian government in 2014 as part of the India Newborn Action Plan (INAP).
- Reliable data on stillbirth epidemiology in India is limited.

**What did the researchers do and find?:**

- Verbal autopsy interviews were conducted for 1,103 stillbirths identified from a sample of 109,689 households covering a 610,514 population, which was representative of the Indian state of Bihar.
- The estimated incidence of stillbirths was 21.2 per 1,000 births in Bihar state, and half of the stillbirths were estimated to have occurred before the onset of labour.
- Significant differences were observed in the delivery-related variables and associated maternal conditions that may influence stillbirths depending on the place of delivery and between antepartum and intrapartum stillbirths.
- Qualitative data highlighted important health-provider interface issues, the knowledge of which can possibly improve the success of interventions aimed at better skilled care at birth and delivery to reduce stillbirths.

**What do these findings mean?:**

- These results provide significant insights from a large number of stillbirths that can facilitate contextualisation of the ground reality to improve the reach of the health system to reduce stillbirths in India.
- The findings of this study are timely and pertinent for strengthening INAP, which aims to improve both the data and interventions for stillbirths.

## Introduction

An estimated 2.6 million third-trimester stillbirths occurred in 2015, and almost all were in low-income and middle-income countries [1]. Despite the overlap between the causes of and effective interventions for stillbirths and neonatal deaths [1–4], the former has largely been missing from the policy agenda as a public health issue until recently with the Every Newborn Action Plan [5,6]. A review identified only 3% of research publications on stillbirths from countries that account for nearly 90% of the stillbirth burden, highlighting that the research gap in stillbirths is greater than the 10/90 gap for worldwide health research [7]. Furthermore, there is a research gap in the understanding of conditions and contexts within which stillbirths occur [3,5,8], and much of the available research focuses on improved intrapartum care [5,9,10].

The burden of stillbirths in India remains unacceptably high with about 590,000 stillbirths in 2015, the largest numbers of stillbirths globally [11]. With the increasing attention on stillbirths worldwide, in 2014, as part of the India Newborn Action Plan (INAP), the Indian government adopted a target of <10 stillbirths per 1,000 births by 2030, the first ever national stillbirth-prevention target [12]. The need for improved data on newborn death and stillbirths is highlighted, and effective tracking of such events is recommended in the INAP [12].

We have recently reported a stillbirth rate of 20 per 1,000 births from a large-scale, population-based household survey of women who had a pregnancy outcome in the last 12 months in the Indian state of Bihar [13]. With a population of 104 million in year 2011, Bihar state is the third most populous state in India, with 11% of it urbanised [14]. Using the previously used cluster-sampling frame [13,15], we conducted a study to estimate the causes of death using verbal autopsy. We report on stillbirths in this paper with 3 major objectives: first, to report detailed stillbirth epidemiology based on verbal autopsy in this population; second, to assess contextual information from the qualitative data regarding the interface with the health system that could allow understanding of missed opportunities within the health system; lastly, based on these data, we make recommendations for the INAP that could better inform the setting of priorities and actions to reduce stillbirths.

## Methods

### Ethics

This study was approved by the Institutional Ethics Committee of the Public Health Foundation of India. The research conformed to the principles embodied in the Declaration of Helsinki. All participants provided written informed consent, and for those who could not read or write, the participant information sheet and consent form were explained by the trained interviewer, and a thumb impression was obtained.

### Study design

The sampling method for clusters is described in detail elsewhere [13,15], and the methods relevant to this report are detailed here. The state of Bihar is divided into 38 districts, each of which is divided into 5 to 27 blocks, giving a total of 342 blocks in the state. Within these 342 blocks, the secondary samplings units (SSUs) were villages in rural areas and urban frame survey blocks in urban areas, as defined by the National Sample Survey Organization [16]. The SSUs with <50 households were combined with an adjacent SSU, and the large, rural SSUs were split into equal-sized segments of 75 to 100 households using natural boundaries. A total of 1,017 SSUs were sampled in proportion to the number of SSUs in each block, using simple random sampling without replacement. Therefore, this multistage stratified random sampling approach to obtain a representative state sample provided a total of 1,017 clusters of about 75 to 150 households across all the 38 districts of Bihar (772 rural and 245 urban clusters).

### Data collection

**Fig 1:**
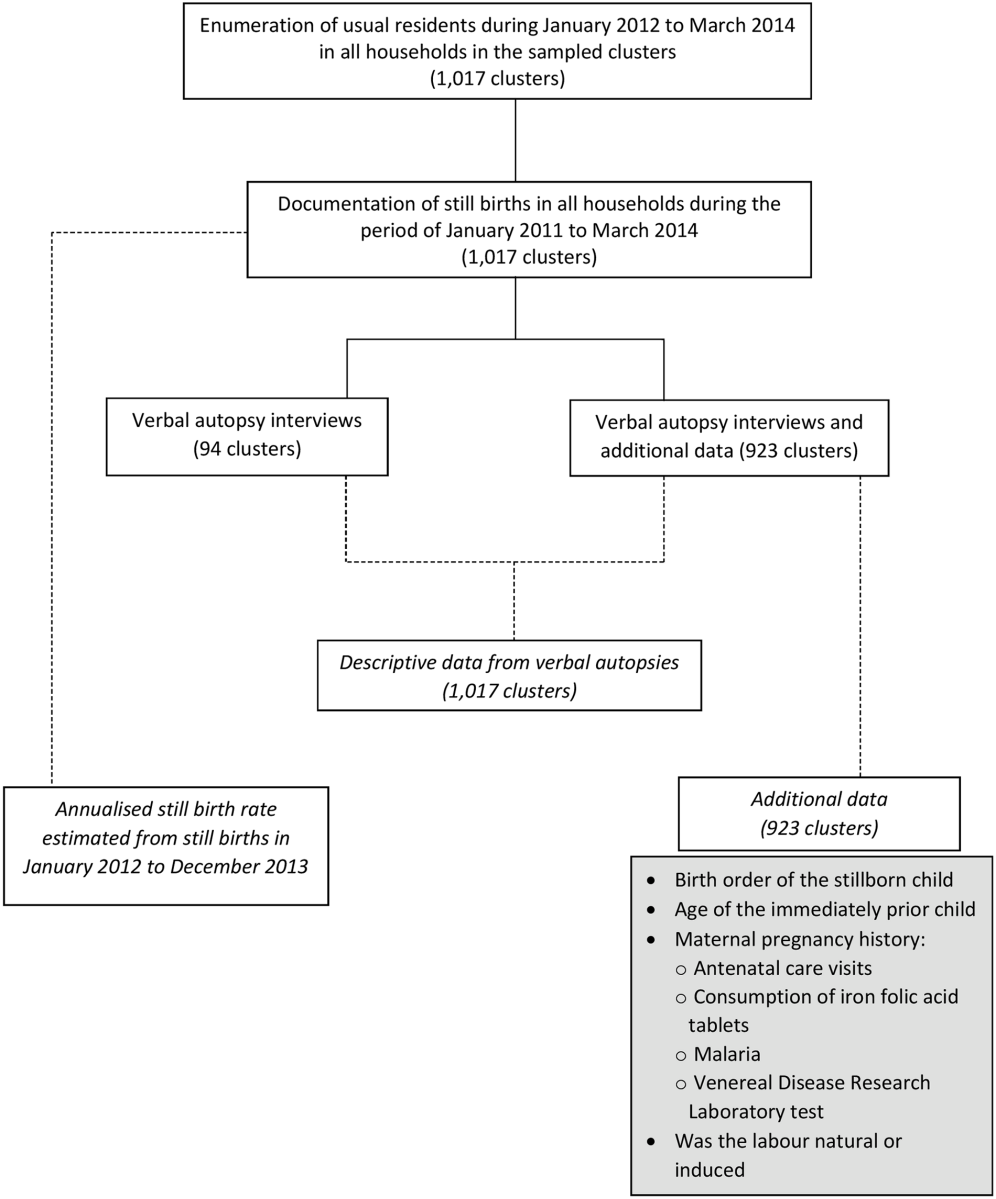
Diagrammatic representation of the data collection process.

This survey was conducted from July 2014 to July 2015. Fig 1 shows the data collection process. In each sampled SSU, all the households (a household was defined as people eating from the same kitchen) were enumerated. During the enumeration, trained interviewers documented the age and sex of all the usual residents in each household of the sampled clusters. Details of members who had in/out-migrated, births, and deaths including stillbirths between January 2012 and March 2014 were collected. In addition, stillbirths in these households from January to December 2011 were additionally documented. During the enumeration, stillbirths were documented by confirming that the baby did not show any sign of life (did not cry, breathe, and move). The permanent and temporary migration (both inwards and outwards), total and of women who were pregnant or had delivery, was documented in detail to ensure that we neither overestimated nor underestimated the total births in this population.

Verbal autopsy interviews were conducted using the Population Health Metrics Research Consortium (PHMRC) shortened verbal autopsy questionnaire designed for neonatal and child deaths (includes stillbirths) [17,18]. For all the stillbirths identified during the enumeration process, after documenting background information including sociodemographic characteristics of the respondents, questions were asked to differentiate between a stillbirth (no sign of life: did not cry, breathe, and move) and a neonatal death that occurred soon after delivery. Specific questions related to stillbirth were subsequently asked only for deaths identified as stillbirth. Close-ended questions documented maternal history during the pregnancy, labour, and delivery, as well as the condition of the baby when delivered. Photographs of macerated and fresh stillborn babies were shown to the respondents to ask questions relating to the stillborn baby’s appearance. This was followed by recording verbatim an open narrative of pregnancy until the delivery of the stillborn baby. The PHMRC questionnaire was translated into Hindi (local language), after which it was back-translated into English to ensure the accurate and relevant meaning and intent of the questions. Pilot testing of the questionnaire was carried out and modifications made as necessary. The interview was conducted using the MS-Access and Open Development Kit software in hand-held tablets; only the verbatim component of the verbal autopsy interview was captured on paper. During the very initial phase of data collection, it was realised that it would be useful to collect additional data, shown in the grey box in Fig 1, which are not captured in the verbal autopsy questionnaire. These additional data on stillbirths were collected in 923 (90%) of the 1,017 clusters.

### Analysis

Before the analysis, all open narratives in the verbal autopsy interviews for the stillbirths were reviewed to confirm that a stillbirth was reported. In addition, we reviewed narratives of neonates who had died on day 0 of birth to check for possible misreporting between neonatal death and a stillbirth. A total of 12 (1.08%) mismatch cases were identified in this review (3 neonatal deaths were reassigned as stillbirths, and 9 stillbirths were reassigned as neonatal deaths). As per the PHMRC protocol, the cause of death was assigned using the validated SmartVA automated algorithm [19–21]. The SmartVA was run on all neonatal and child deaths including stillbirths, and the stillbirths identified using this run were used in this analysis. A stillbirth was defined as a foetal death with a gestation period of ≥28 weeks wherein the foetus did not show any sign of life (did not cry, breathe, and move). Data on gestation period were reported in months by the respondents, as is the practice in India, which was converted into weeks for analytical purpose (1 month = 4 weeks).

We report the annualised stillbirth incidence estimate for the state of Bihar using two years of data from January 2012 to December 2013 by considering the proportion of stillbirths from the total births including stillbirths for this given time period (Fig 1). We present detailed data on stillbirths using all cases documented from 1 January 2011 to 31 March 2014 (Fig 1). An attempt was made to understand stillbirths as antepartum and intrapartum deaths using the questions regarding the baby’s last movement felt by the mother to assess the time of death and the description of the stillborn baby documented as fresh or macerated. An antepartum death is a death before the onset of labour as evidenced by either maceration of the stillborn or by a report of loss of baby’s movement before the onset of labour even without maceration [22]. A death after the onset of labour, as evidenced by lack of maceration and by reporting of baby’s movements after the onset of labour, was considered an intrapartum death (fresh death) [22]. In the PHMRC verbal autopsy questionnaire, the baby’s last movement felt by the mother is documented as hours prior to the delivery. We followed a previously used cutoff of within 8 hours since the last-felt baby’s movement to classify the stillbirth as fresh, and macerated was considered for the last baby movement felt at least 8 hours or more before the delivery [23]; this cutoff was based on the most commonly used maceration criteria [24], and it is also in guidelines for perinatal autopsy [25]. Considering the mixed reports on relying on a stillborn baby’s appearance using verbal autopsy [23,26,27], we gave preference to the baby’s movement over the description of the stillborn baby.

To ascertain possible underlying risk factors for stillbirth, we used the responses to the question regarding “complications during the last 3 months of pregnancy”; the complications documented in the PHMRC verbal autopsy questionnaire are: maternal conditions (convulsions, high blood pressure, severe anaemia, or diabetes during the last 3 months of pregnancy, or fever during labour), congenital anomaly, obstructive delivery (breech position of the baby, cord delivered first, or cord entangled around the baby’s neck), and excessive bleeding during delivery. The underlying risk factor for stillbirth was considered unknown if none of these complications were reported.

For the second objective, we reviewed the open-ended narrative in the verbal autopsy questionnaire within the context of interface proposed to increase the reach of the existing health system [4]. The aim of this review was to assess patterns that could facilitate the understanding of avoidability or missed opportunities that could guide reduction of mortality due to stillbirth. Four team members (AK, PS, SG, MA) reviewed 50 open narratives each and noted the variety of information available from these reviews. These 200 narratives were then used to form thematic areas under which the narratives were to be classified. The team members exchanged and reviewed 50 narratives each to classify. These 200 classifications were reviewed with RD, and modifications were made as needed. This review system was then applied to the remaining narratives; all narratives were reviewed by at least 2 team members. Given that the open-ended format does not allow computation of numbers, we present case studies across the major themes identified to highlight action areas that need attention.

Other than SmartVA, the rest of the analyses were performed using STATA 11.2 software (Stata Corp, USA). Ninety-five percent confidence intervals (CI) are reported for the stillbirth rate. Chi-square test is reported where relevant to assess significant associations. This study did not have a predefined, written prospective analytical plan. The analyses that reported on stillbirth incidence and description were decided prior to data collection, and analysis of the qualitative review of open narratives was planned after data collection, with some of the preliminary results contributing to the further examination of emerging themes in this review. The study findings are reported as per STROBE (S1 STROBE Checklist) and COREQ (S1 COREQ Checklist) checklists.

## Results

### Stillbirth incidence

A total of 1,132 stillbirths were identified from January 2011 to March 2014 from 109,689 households (87.1% participation) covering a population of 610,514. Verbal autopsy interviews were available for 1,103 (97.4%) stillbirths; of these, 790 (71.6%) were between January 2012 and December 2013. With 19,692 total births in this population between January 2012 and December 2013, the estimated annualised incidence of stillbirths was 21.2 (95% CI 19.7 to 22.6) per 1,000 births for the state of Bihar. This was 22.6 (95% CI 20.9 to 24.2) and 15.4 (95% CI 12.6–18.2) per 1,000 births for rural and urban areas of the state, respectively.

### Stillbirth descriptive data

The respondent for verbal autopsy was the mother in 76.8% of the interviews, followed by the grandmother (11.9%). The reported gestation period distribution for the 1,103 stillbirths was as follows: 7 months (13.1%), 8 months (12.7%), 9 months (67.2%), and 10 months (4.6%)—this information was not available for 1 case. Overall, 142 (12.9%) of the stillborn babies were delivered by caesarean section. Basic characteristics for 1,103 identified stillbirths, by place of residence, are shown in Table 1. Among 1,103 stillborn babies, 686 (62.2%) were boys, 327 (29.7%) were firstborn, and 760 (68.9%) were delivered at a health facility. The only significant difference seen in rural and urban residence settings was that a significantly higher proportion of mothers in the former setting had their baby delivered by an unskilled health provider (28.4%) than in the latter setting (*p* = 0.007).

**Table 1:** Basic descriptive data for births that resulted in a stillbirth between January 2011 and March 2014 in the Indian state of Bihar.

Characteristic	Urban residence *N* = 170 (% of total)	Rural residence *N* = 933 (% of total)	Chi-square test of significance	Total *N* = 1,103 (% of total)
Stillborn baby was a boy	104 (61.2)	582 (62.4)	*p* = 0.063	686 (62.2)
Singleton birth	165 (97.1)	887 (95.1)	*p* = 0.256	1,052 (95.4)
Stillborn baby was firstborn*‡	50 (29.4)	277 (29.7)	*p* = 0.845	327 (29.7)
Maternal age between 18–29 years*	122 (71.8)	551 (59.1)	*p* = 0.009	673 (61.0)
Maternal age between 30–35 years*	30 (17.7)	240 (25.7)	*p* = 0.009	270 (24.5)
Immediately prior child aged 2 years or less*‡	24 (18.8)	116 (16.1)	*p* = 0.467	140 (16.5)
Had at least 1 antenatal visit during the pregnancy*‡	103 (60.6)	513 (55.0)	*p* = 0.036	616 (55.9)
Health facility delivery	120 (70.6)	640 (68.6)	*p* = 0.332	760 (68.9)
Vaginal delivery*	139 (81.8)	795 (85.2)	*p* = 0.332	934 (84.7)
Unskilled health provider delivered the baby†	32 (18.8)	265 (28.4)	*p* = 0.007	297 (26.9)

*Data were not available for 254 stillbirths on birth order, 88 on maternal age, 25 on age of the previous child, 213 on antenatal visits, and 27 on vaginal delivery

^†^Includes dais, auxiliary nurse midwife, and family members/relatives/friends; Data were not available for 120

^‡^Variables derived from an additional questionnaire

**Fig 2:**
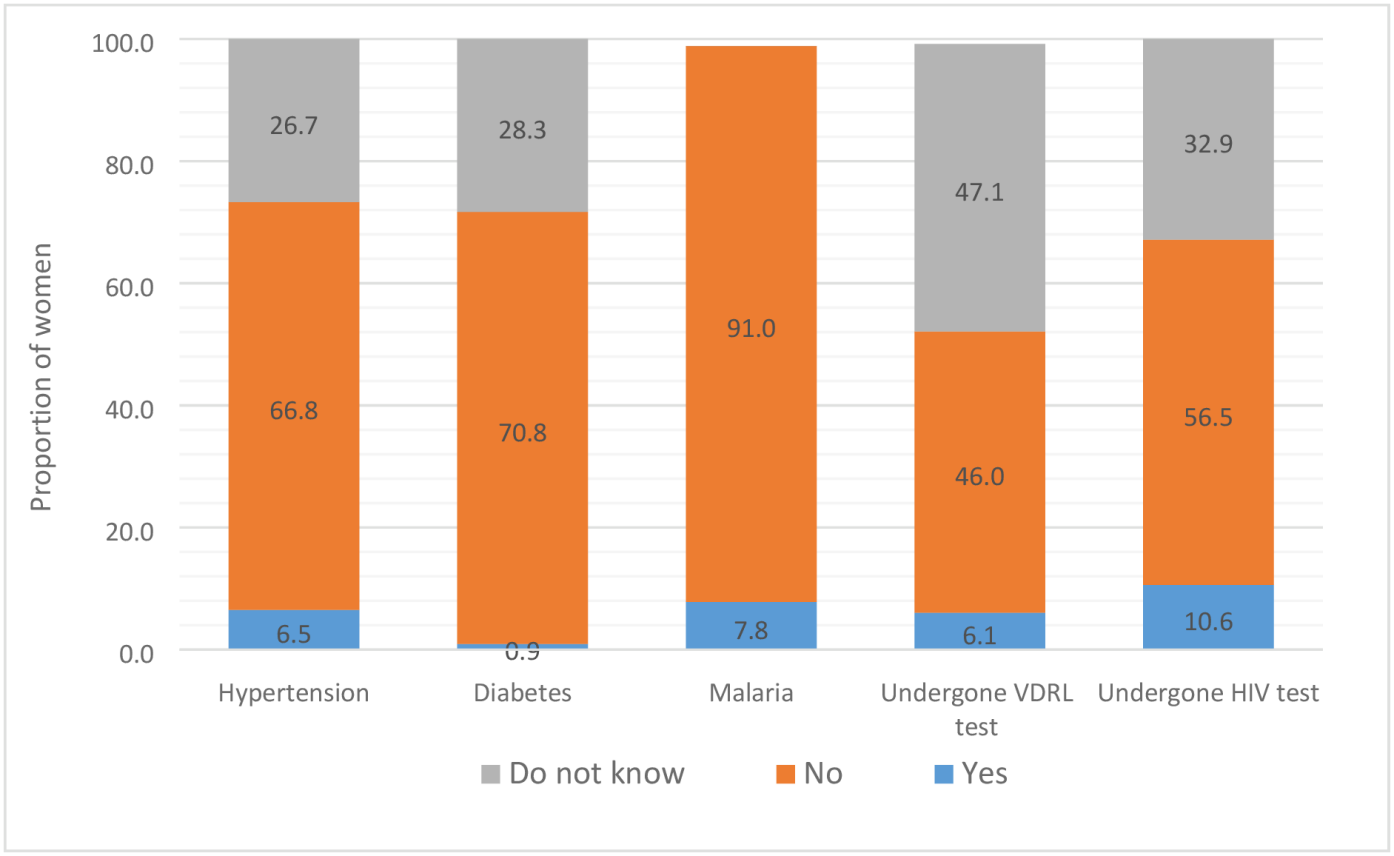
The distribution of select self-reported associated maternal conditions that had resulted in stillbirth and diagnostic tests performed during pregnancy in the Indian state of Bihar. VDRL test, Venereal Disease Research Laboratory test.

Table 2 documents the distribution of delivery-related variables and associated maternal conditions by place of delivery. Some significant differences were seen based on the place of delivery. Spontaneous labour (79.2%) and small/smaller size of the baby (38.1%) were reported for a significantly (*p* < 0.001) higher proportion of stillbirths in home delivery (341) as compared with the health facility deliveries. On the other hand, significantly (*p* < 0.001) more women who had delivered in a health facility reported antenatal care visits (59.3%), and fewer of them reported not consuming iron folic acid tablets (28.6%). Reporting of the associated maternal conditions and diagnostic tests was poor (Fig 2). A total of 26.7% and 28.3% respondents reported “do not know” for hypertension and diabetes during pregnancy. Nearly half of the respondents reported “do not know” if they had undergone a VDRL (Venereal Disease Research Laboratory) test to rule out syphilis (47.1%), and one-third responded “do not know” for having undergone a HIV test (32.9%) during pregnancy.

**Table 2:** Variation in the distribution of delivery-related variables and associated maternal conditions for stillbirths between January 2011 and March 2014 in the Indian state of Bihar. Twenty-six deliveries that occurred en route to the hospital are included under health facility delivery. Place of delivery was not available for 2 cases.

Variable	Health facility delivery *N* = 760 (% of total)	Home delivery *N* = 341 (% of total)	Chi-square test of significance	Total *N* = 1,101 (% of total)
Delivery detail				
Mother reported movement of the baby <8 hours before delivery	295 (38.8)	149 (43.7)	*p* = 0.125	444 (40.3)
Spontaneous labour†#	400 (52.6)	270 (79.2)	*p* < 0.001	670 (60.9)
Foul-smelling liquor†	156 (20.5)	78 (22.9)	*p* < 0.001	234 (21.3)
Doctor/nurse delivered the baby†	645 (84.9)	39 (11.4)	*p* < 0.001	684 (62.1)
Vaginal delivery†	601 (79.1)	332 (97.4)	*p* < 0.001	933 (84.7)
Entangled cord	50 (6.6)	23 (6.7)	*p* = 0.919	73 (6.6)
Breech position of the foetus	97 (12.8)	42 (12.3)	*p* = 0.837	139 (12.6)
Baby was very small/small in size†	189 (24.9)	130 (38.1)	*p* < 0.001	319 (29.0)
Baby was of average size†	383 (50.4)	162 (47.5)	*p* < 0.001	545 (49.5)
Baby’s skin and tissue pulpy	180 (23.7)	82 (24.1)	*p* = 0.015	262 (23.8)
Maternal issues/conditions				
Had at least 1 antenatal visit†#	451 (59.3)	164 (48.1)	*p* < 0.001	615 (55.9)
Did not consume iron folic acid tablets†#	217 (28.6)	150 (44.0)	*p* < 0.001	367 (33.3)
No report of pregnancy complications of interest‡	364 (47.9)	177 (51.9)	*p* = 0.218	541 (49.1)
High blood pressure	61 (8.0)	20 (5.9)	*p* < 0.001	81 (7.4)
Anaemia	172 (22.6)	60 (17.6)	*p* = 0.058	232 (21.1)
Fever during labour	111 (14.6)	56 (16.4)	*p* = 0.437	167 (15.2)
Diabetes	5 (0.7)	3 (0.9)	*p* < 0.001	8 (0.7)
Convulsions	13 (1.7)	7 (2.1)	*p* = 0.694	20 (1.8)
Malaria*#	44 (5.8)	24 (7.0)	*p* = 0.075	68 (6.2)
Mother smoked tobacco*#	16 (2.1)	9 (2.6)	*p* = 0.068	25 (2.3)

*Data not available for 253 on malaria and 251 on mother smoking tobacco

^†^Data were not available for 148 stillbirths on spontaneous labour, 26 on foul-smelling liquor, 120 on doctor/nurse delivered the baby, 27 on vaginal delivery, 91 on size of the baby, 213 on had at least 1 antenatal visit, and 243 on iron folic acid tablets

^‡^Convulsions, high blood pressure, severe anaemia, diabetes, and fever during labour

^#^Variables derived from an additional questionnaire

**Fig 3:**
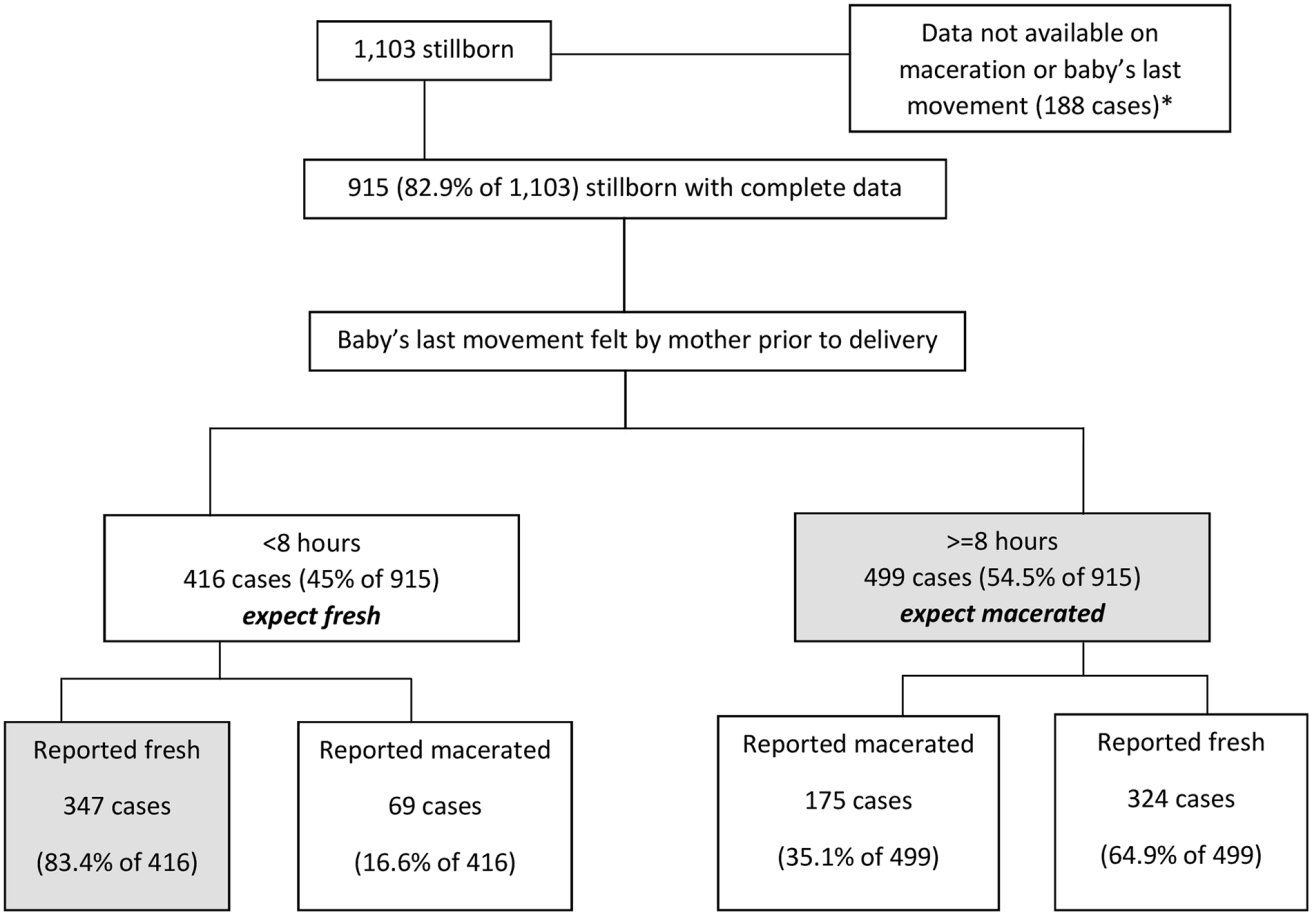
The classification process used to identify antepartum and intrapartum deaths for the stillbirths in the Indian state of Bihar. *Skin appearance and the baby’s movement were reported as “do not know” for 96 and 116 cases, respectively. For an additional 24 cases, both were reported as “do not know”.

Fig 3 summarises the classification process used to identify antepartum and intrapartum deaths. For the 915 stillborn babies (82.9%) for whom data both on the baby’s movement felt by the mother before delivery and skin maceration were available, 499 (54.5%) were considered as antepartum and 347 (37.9%) as intrapartum deaths, and the rest (7.6%) could not be classified. The reported mismatch between the baby’s movement and appearance is very apparent with these data, and this mismatch was more pronounced for antepartum deaths wherein fresh appearance was reported for 64.9% of the cases (Fig 3). Had we given preference to the description of the stillborn baby over the baby’s movement, then 244 (23.8%) cases of stillbirth would be classified as antepartum and 671 (67.4%) cases as intrapartum deaths. Some significant differences were seen in the distribution of delivery-related variables and associated maternal conditions for the 846 stillbirths that could be classified as antepartum or intrapartum deaths (Table 3). A significantly higher proportion of foul-smelling liquor (30.9%; *p* < 0.001), history of maternal anaemia (24.9%; *p* = 0.002), and fever during labour (19.4%; *p* < 0.001) were reported for antepartum deaths as compared with intrapartum deaths (Table 3).

**Table 3:** Variation in the distribution of delivery-related variables and associated maternal conditions based on type of stillbirth for stillbirths between January 2011 and March 2014 in the Indian state of Bihar. Type of stillbirth was classifiable for 846 stillbirths.

Variable	Antepartum *N* = 499 (% of total)	Intrapartum *N* = 347 (% of total)	Chi-square test of significance	Total *N* = 846 (% of total)
Delivery details				
Spontaneous labour*#	280 (56.1)	227 (65.4)	*p* = 0.010	507 (59.9)
Foul-smelling liquor†	154 (30.9)	43 (12.4)	*p* < 0.001	197 (23.3)
Doctor/nurse delivered the baby*	318 (63.7)	198 (57.1)	*p* = 0.144	516 (61.0)
Vaginal delivery*	435 (87.2)	302 (87.0)	*p* = 0.231	737 (87.1)
Entangled cord	29 (5.8)	27 (7.8)	*p* = 0.257	56 (6.6)
Breech position of the baby	69 (13.8)	41 (11.8)	*p* = 0.392	110 (13.0)
Baby was very small/small in size*	167 (33.5)	88 (25.4)	*p* = 0.011	255 (30.1)
Maternal issues/conditions				
Had at least 1 antenatal visit*#	291 (72.4)	187 (64.5)	*p* = 0.045	478 (69.1)
Did not consume iron folic acid tablets*#	182 (36.5)	115 (33.1)	*p* = 0.346	297 (35.1)
No report of pregnancy complications of interest‡	212 (42.5)	194 (55.9)	*p* < 0.001	406 (48.0)
High blood pressure	43 (8.6)	21 (6.1)	*p* = 0.312	64 (7.6)
Anaemia	124 (24.9)	55 (15.9)	*p* < 0.001	179 (21.2)
Fever during labour†	97 (19.4)	36 (10.4)	*p* < 0.001	133 (15.7)
Diabetes	4 (0.8)	3 (0.9)	*p* = 0.985	7 (0.8)
Convulsions	9 (1.8)	6 (1.7)	*p* = 0.936	15 (1.8)
Malaria*#	34 (6.8)	24 (6.9)	*p* = 0.628	58 (6.9)
Mother smoked tobacco*#	15 (3.0)	7 (2.0)	*p* = 0.433	22 (2.6)

*Data were not available for 116 on spontaneous labour, for 95 on doctor/nurse delivered the baby, for 4 on vaginal delivery, for 65 on baby was very small, for 154 on had at least 1 antenatal care visit, for 180 on iron folic acid tablets, for 187 on malaria, and for 188 on mother smoked tobacco

^†^Data were not available for 3

^‡^Convulsions, high blood pressure, severe anaemia, diabetes, fever during labour

^#^Variables derived from an additional questionnaire

**Fig 4:**
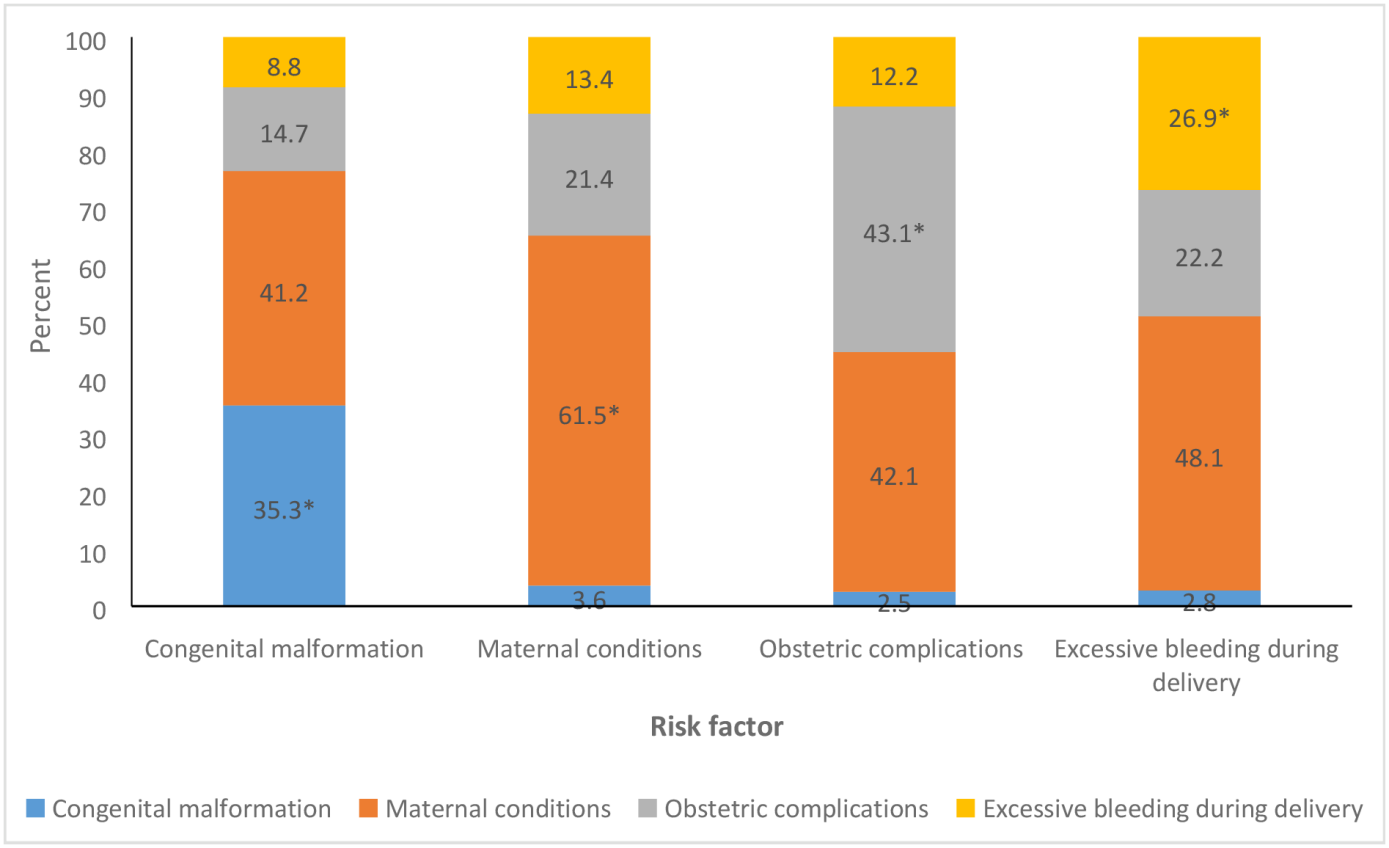
The distribution of the overlap between the various possible risk factors for stillbirth in the Indian state of Bihar. Each bar denotes a risk factor indicated on the *x*-axis, and the stacks in each bar denote the overlap with the other risk factors. *Denotes no overlap with another risk factor.

Among the 1,103 stillborn, no maternal complications during the last 3 months of pregnancy were reported in 377 (34.2%) cases; hence, these were treated as unexplained stillbirths. The possible underlying risk factors for stillbirth were (not mutually exclusive) congenital malformations in 34 (3.1%), maternal conditions in 387 (35.1%), obstetrics complications in 197 (17.9%), and excessive bleeding during delivery in 108 (9.8%) cases. Among the 197 cases of obstetrics complications, breech position was reported in 139 (70.6%), cord entangled around the baby’s neck in 73 (37.1%), and cord delivered first in 14 (7.1%) cases. Fig 4 shows the overlap between the various possible causes of stillbirth. The most overlapping condition reported was maternal conditions, ranging from 40% to 50%. The 387 cases of maternal conditions included (not mutually exclusive) anaemia (60%), fever during labour (43.7%), hypertension (20.1%), convulsions (5.2%), and diabetes (2.1%).

### Qualitative review of verbal autopsy narratives

Broadly, 5 themes emerged based on the review of the open narratives: lack of timely attention by a healthcare provider, poor skills of the healthcare provider (knowledge or implementation), reluctance by the healthcare provider to deliver the dead baby, family-related issues (community), and unknown reason. The following case studies from the verbal autopsy narratives exemplify these themes/scenarios (translated verbatim as is).

### Theme: Lack of timely attention by the healthcare provider

#### Case report 1

Maternal age: 35 years of age; respondent: mother

Mother was full-term pregnant when she participated in a wedding at which she danced and sang. She felt unwell after this episode and took some medication. Her health condition worsened over the next 3 days, during which she felt that she may die. Family members took her to the government hospital where she waited for long time to see the doctor. Her condition deteriorated further during the wait, so the family members decided to take her to a private hospital. A doctor checked her and informed her that the baby was dead inside the womb. He advised her to undergo surgery to remove the dead baby, but the family insisted on a normal delivery. The mother’s condition deteriorated significantly during delivery, but she survived.

#### Case report 2

Maternal age: not known; respondent: grandmother

The mother was full-term pregnant. One night she reported sudden pain in her stomach, and the family took her to the hospital. She kept waiting for the doctor to see her, and the pain increased considerably during the wait. She was able to feel the baby move when she was waiting at the hospital. She then delivered a dead baby. The family reported that the baby died because of a lack of timely attention from the doctor.

### Theme: Poor skills of the healthcare provider

#### Case report 3

Maternal age: unknown; respondent: grandmother

The mother was full-term pregnant and was taken for a routine examination where everything was reported to be fine. About 5 hours postexamination, labour pains started, following which she was taken to the government hospital. During the 2 hours of the labour/delivery process, scissors hit the head of the baby, and there was bleeding on the baby’s head. When the baby was pulled out, there was active bleeding on the head and the baby did not breath, cry, or move. The baby was born dead.

#### Case report 4

Maternal age: 25 years; respondent: grandmother

The baby was born dead. The mother was full-term pregnant. She was taken to the government hospital when the labour pains started. Her mother-in-law went in for delivery with the nurse. The baby girl’s legs came out first, and before the neck could come out, the pain stopped. Then, the nurse tried to pull the baby out, and once the baby was out, she was born dead. The mother thinks that if she were operated upon to take the baby out instead of pulling the baby, her baby would have been alive.

#### Case report 5

Maternal age: 18 years; respondent: mother

The baby was born dead because the cord was entangled around the baby’s neck. When the baby was being delivered, the baby’s legs and hands came out first, and the head was not coming out. Nearly 4 hours into this process, the mother defecated and urinated during the delivery, and she assumed that it was her bowel movement/urine that killed her child. However, the doctor told her that the baby died because of the entangled cord. He also told her that she may not be able to have a baby for the next 5 years because inside of her abdomen was affected during this delivery.

#### Case report 6

Maternal age: 25 years; respondent: grandmother

The mother-in-law informed the interviewer that her daughter-in-law had abdominal pain during the entire pregnancy. She was being treated for this pain at a private hospital. When the labour pains started, she also had a slight fever. When she was taken to the same hospital where she was undergoing treatment, the nurse checked her and said the delivery was not due yet and asked her to come back later. The next day, the pain increased, and she was taken again to the same hospital where she was admitted for 2 days. The labour pains continued, and the baby was delivered on the third morning. The baby was born dead and was also weak. The respondent thinks that the baby died because of prolonged and significant pain.

### Theme: Lack of timely attention by the healthcare provider/poor skills of the healthcare provider

#### Case report 7

Maternal age: 20 years; respondent: mother

According to the mother, she went to a private hospital for a check-up during the third month of pregnancy. The doctor checked her blood and urine and said all was OK. He prescribed some tablets to her. She ate those tablets for 2 months but discontinued as she felt uncomfortable after consuming those tablets. Then, she went for the next check-up in the seventh month of pregnancy; the doctor checked her with a stethoscope and said everything was ok. When she completed her ninth month of pregnancy, she was feeling the baby move. When her labour pain started, she was taken to the government hospital by the accredited social health activist (ASHA) and was informed that the doctor would come in half an hour. However, the pain increased, and she became very uncomfortable, so she decided to go to a private hospital. There, the doctor gave her IV fluids and injection, but still the baby was not delivered. So, the doctor referred her back to the government hospital. The doctor in the government hospital said that baby will not be born soon, and so she moved back to the private hospital. That doctor again gave her injections, and because the baby was still not delivered, he decided to do an operation to take the baby out. The baby was born dead. The doctor informed the mother that the baby was dead already inside the womb, and the mother saw some injury signs on the baby’s head.

### Theme: Reluctance by a healthcare provider to deliver the dead baby

#### Case report 8

Maternal age: 32 years; respondent: mother

According to the mother, during the pregnancy, she had a low-grade fever and anaemia, which caused her to feel weak all the time. In the eighth month of pregnancy, she had labour pains. She applied some medicine and felt better with massage. Around 10 PM to 11 PM, the water broke, and the family called the village Dai (unskilled birth attendant). The Dai suggested that the mother should be taken to the hospital, and she was taken there the same night. The doctor checked her and informed her that the baby was dead in the womb. He referred her to another hospital for delivery as he did not want to deliver a dead child. After many requests, the doctor agreed to deliver the dead baby around 1.30 AM.

#### Case report 9

Maternal age: 34 years; respondent: mother

The mother informed the interviewer that she was in her 10th month of pregnancy. She went to see the doctor who told her that the baby had been dead in the womb for at least 1 day. He then asked her to go to another hospital for delivery as he did not want to deliver a dead child. The mother requested for delivery at the initial hospital as the other hospital was quite far. The doctor refused. After considerable appeal, he agreed. The baby was born dead and was big in size.

### Theme: Family-related issues

#### Case report 10

Maternal age: 29 years; respondent: mother

The baby was born dead. Sometime during the ninth month of pregnancy, when the mother stopped feeling the baby’s movement, she informed her family members and asked to be taken to the hospital. They refused. She insisted and then went on her own. The doctor checked and informed her that the baby had been dead in the womb for at least 2 days. He mentioned that had she come to the hospital earlier, perhaps the baby would have been alive. The mother thinks that the baby died because of the negligent attitude of her family.

### Theme: Unknown reason

#### Case report 11

Maternal age: 43 years; respondent: mother

Four months into the pregnancy, the mother reported watery discharge and pain. She was seen by a doctor who tested her blood and urine and then prescribed some medicines. The doctor said that the baby was fine. The mother became healthier with the use of the medicines. When she had completed 9 months of pregnancy, around 4 PM she felt pain but could not feel the baby move. She was taken to the government hospital at 5 AM the next morning where the nurse delivered her baby by normal procedure. The baby did not cry, breathe, or move. The nurse kept the baby in an incubator, and still the baby did not breathe. The baby was taken out and given to the family. The mother thinks that the baby stopped moving around 12 hours before delivery.

#### Case report 12

Maternal age: 20 years; respondent: mother

The baby was born dead in the ninth month of pregnancy at the government hospital. The mother’s water broke, but she had no labour pains. She was taken to the hospital where the pain was induced by an injection. The baby was delivered normally but did not cry, breathe, or move. The doctor declared the baby dead. One year prior, the mother had lost another baby like this.

## Discussion

To our knowledge, this study is among the few large, population-based assessments of stillbirths using verbal autopsy interviews at state level in India. The annualised incidence of stillbirths was 21.2 per 1,000 births for the state of Bihar, with it being higher in the rural areas of the state, and half were estimated to be antepartum. Some significant differences were seen in delivery-related variables and associated maternal conditions based on the place of delivery and type of stillbirth. Review of the open narrative highlighted the acute need for documenting more relevant information for stillbirths that can further assist with the reduction of stillbirths.

Intrapartum death rates are frequently used as an indicator of quality of care, as most intrapartum stillbirths are associated with potentially preventable complications that arise during labour [10,28,29]. Given that we found intrapartum deaths less often than antepartum deaths, it could imply that there is a certain level of obstetrics care available in this population. However, it is to be noted that the care available was suboptimal, as breech position and the cord becoming entangled around the baby’s neck made up most of the obstetrics complications that resulted in a stillbirth. In addition, the open narratives highlighted suboptimal care with “push and pull” performed during the delivery and the healthcare provider having “no prior knowledge of breech position of the baby” before delivery. Emergency obstetric care including caesarean section is known to be highly effective to reduce stillbirths [2,10]. Nearly 15% of the stillborn babies were delivered by caesarean section in our sample, and though it is not possible to assess the need for caesarean section from the verbal autopsy questionnaire, review of open narratives highlighted that some health providers in obstructed deliveries and/or prolonged labour continued to wait for a vaginal delivery instead of opting for a timely caesarean section that could have possibly reduced the chance of stillbirth in some cases [10,30]. Though improved skilled care at birth and delivery is the key to reduce intrapartum deaths, these data suggest that more is needed to improve the skills of healthcare providers. Seventy percent of the women in this study delivered in a health facility, a proportion similar to that reported in a representative survey of women with live births in 2013 in the same population [31]. Importantly, the open narrative reviews highlight that mere delivery in a health facility or presence of a skilled health provider does not guarantee skilled delivery care of a reasonable quality. Of the 5 major themes identified in open narratives, 3 were related to healthcare providers—lack of timely attention, poor skills (knowledge or implementation), and reluctance to deliver a dead baby. With major activities proposed under the INAP to improve quality of care during labour and childbirth being centred around the health providers [12], it is important to document and monitor health provider interface that can possibly improve the success of interventions aimed at improving skilled care at birth and delivery. In order to do so, the INAP should consider examination of individual stillbirth cases to identify underlying reasons for these deaths, so that opportunities are provided from these learnings to prevent similar deaths in the future. Guidelines for such an audit of stillbirth are readily available [32].

Most stillbirths are antepartum deaths, irrespective of the global setting [7,33,34], which is also true for this study population. Adequate antenatal care is an effective intervention to reduce stillbirths by preventing, identifying, or treating pregnancies with likely adverse outcomes due to infections, maternal conditions such as hypertension and diabetes, and malnutrition [2,35]. Only a little over half of the women who had a stillbirth reported at least 1 antenatal care visit in our study. In comparison, in a representative survey of women with a live birth in 2013 in the same population, 82.8% of the women reported at least 1 antenatal visit [31], suggesting that antenatal care provides a natural contact with a healthcare provider through which requisite interventions for the pregnant women can be facilitated [35]. As poor retention of women in antenatal care services and inequity in access to antenatal care services has been previously documented in this population [15], ensuring improved coverage and equity in antenatal care services is imperative for the proposed priority antenatal care interventions under the INAP to reduce stillbirths [12]. We strongly recommend that this be one of the stated goals of INAP. The proportion of women who reported consuming iron folic acid tablets (irrespective of the quantity) was similar in our study (41.9%) and in a previous household survey of women with live births (46%) [31]. Periconceptional folic acid fortification is an effective intervention against neural tube defects resulting in stillbirths [2]; however, the available data do not allow us to comment on the use of iron folic acid before conception in our study population. In this study, 62% of the stillborn babies were reported to be boys. A recent meta-analysis based on more than 30 million birth outcomes reported a 10% elevated risk of stillbirth in boys as compared with girls [36]. It is important to note that this meta-analysis showed equal ratios of stillbirth in boys and girls for India, based on data from 1 state in southern India. The larger disparity in stillbirths by gender in our study could partly be a reflection of son preference, which has resulted in a sex ratio imbalance in India due to female foeticide [37,38].

Syphilis, malaria, and other infections, placental conditions, congenital anomalies, and pregnancy-induced hypertension cause the majority of antepartum deaths globally [39,40]. In fact, syphilis and malaria are the main infectious causes of third-trimester stillbirths in low-income countries [39,41,42]. Only 6.1% of the women reported having undergone the VDRL test to rule out syphilis, and 7.8% reported suffering from malaria during pregnancy. We did not document the results of VDRL tests. It is important to point out that 46% of the respondents said that the VDRL test was not performed, and a similar proportion did not know if the VDRL test was performed or not. Under the antenatal care guidelines recommended by the Health Ministry in India, a test to rule out syphilis is considered desirable but not mandatory [43]. Rapid Plasma Reagin test is also recommended to test for syphilis in addition to VDRL test in these guidelines [43]; however, we did not document it. Though a significant decrease in maternal and congenital syphilis from 2008 to 2012 in India was suggested by a recent modelling [44], several limitations of the data used have been highlighted [44,45] and recommendations made to strengthen syphilis surveillance in India [45].

The current system of verbal autopsy does not provide much information on the possible underlying risk factors for stillbirth [7,26,46]. We used the responses to the question regarding “complications during the last 3 months of pregnancy” to ascertain possible underlying risk factors for stillbirth. For one-third of the stillbirths, the possible risk factors were unexplained. Two-thirds of the women with stillbirth reported at least 1 complication during the last 3 months of pregnancy, with maternal conditions including anaemia, fever during labour, and hypertension accounting for most of the complications. Notably, the maternal conditions overlapped quite significantly with the other possible underlying risk factors for stillbirth. Obstetrics complications and excessive bleeding during delivery contributed to nearly 30% of the cases as possible risk factors for stillbirth, again, highlighting the need for improved skilled care during delivery. Similar risk factors for stillbirth have been reported previously from an Indian population [47].

The study findings should be interpreted within the following limitations. We defined stillbirth based on the gestation period and not based on birth weight, as is done in several studies in a developing country setting [7]. Capture by birth weight yields lower stillbirth rates than the gestational age, and the latter is a better predictor of maturity than the former [7,11]. Birth weight for stillborn babies in our study was not available for 84.5% of the stillborn babies; this is not unusual, as unrecorded birth weight for all births (dead and alive) was reported at 57.1% in the latest round of the Annual Health Survey in Bihar [48]. Though the size of babies at birth is not an accurate depiction of birth weight, half of the stillborn babies in this study were reported to be of average size, and 29% were reported to be small/very small in size. We captured the gestational age in months and converted it into weeks for this analysis. Culturally, pregnancy length in India is reported in months and, for many, is based on the last menstrual period, which is considered a reliable estimate for measuring gestational age in both developing and developed country settings [49,50]. With a mean difference of 1 to 1.3 days in length of pregnancy reported between the last menstrual period and other methods [49,50], we believe that this would not result in a significant difference in our estimates of stillbirths. We considered a previously used cutoff of within 8 hours since the last-felt baby’s movement to classify stillbirths as antepartum or intrapartum [23–25]. The baby’s movement was preferred over the description of the stillborn baby to classify stillbirths, as appearance is reported to be a less accurate proxy for death-to-delivery interval both by community and health providers because maceration is a subjective diagnosis, with the skin changes ranging from minimal to extensive [23,26,27]. In addition, several other factors such as high microbial load in the amniotic fluid, long duration of hypoxia prior to actual death, and maternal fever may contribute to more maceration than would otherwise be expected for a certain death-to-delivery interval [23]. Because peeling skin is associated with death of at least 8 hours [24], it is used as a cutoff for the perinatal and paediatric autopsy [25]. Our data further contribute to the evidence of significant mismatch in stillbirth classification when a stillborn baby’s appearance is taken as a predictor of death-to-delivery interval [23,27]. To improve the estimates of antepartum and intrapartum stillbirths, we recommend that INAP should encourage the documentation of accurate time of delivery and time of the baby’s demise when possible through foetal heart rate monitoring in medical records at health facilities, which could provide critical data to focus efforts towards measuring and improving stillbirth outcomes in India.

There are several strengths of this study. These, we believe, are the first large-scale representative data on stillbirth from India that provide detailed epidemiology. Our effort to get the total births as accurate as possible by documenting all in/out migration among the reproductive-age women who had a pregnancy outcome in the period of interest provides an appropriate denominator for the stillbirth estimation. Furthermore, we confirmed stillbirth at 3 points in time from enumeration through the analysis, thereby strengthening the numerator for these estimates. This approach is different from the Demographic and Health Surveys (DHS), which rely only on the documentation of stillbirth as informed by the respondent without confirming any sign of life [51–53]. The descriptive data documented in addition to verbal autopsy on the associated maternal conditions is valuable in prioritising interventions that could benefit reduction in stillbirths, as these are not routinely tracked at population level [3]. The review of the open narratives provides an added value for the context of stillbirths. Given that these were open-ended narratives, and not all information/themes were covered across all the interviews, these highlighted several documentable themes that can facilitate contextualisation of the ground reality to improve the reach of the existing health system to reduce stillbirths. Lastly, this study adds to the research priorities identified for stillbirth epidemiology [3].

The stillbirth incidence rate in this population is similar to the modelled estimate of stillbirth incidence at 22 per 1,000 births for India [54]. This estimate is modelled because of the poor availability of high-quality data on stillbirths in India and underreporting of stillbirths, including in the Sample Registration System and the DHS [12,54]. The registration of stillbirths has been suggested to improve data on stillbirths; however, various opportunities and challenges related to registration have also been identified [7]. In the Indian health system, there is also a challenge of accountability in the documentation of stillbirths and neonatal deaths, as it is known that the health providers often prefer to document neonatal deaths that occur immediately postbirth as stillbirths instead of as neonatal deaths to deflect the accountability of an unsuccessful attempt to save a life. However, the INAP offers a choice for India to improve its approach to documentation of the national stillbirth estimates and to make a reasonable difference in stillbirths. The INAP aims to establish a sound surveillance system for tracking stillbirths, and it has suggested several priority actions to address the bottlenecks in the health system, including the validation of routine data collected in the health facilities, quality and training of human resources, quality of care, leadership and governance, and financing to reduce stillbirths [12]. It is imperative that documentation in medical records improve including data on timing of delivery and foetal heart rate monitoring in order to have a more reasonable understanding of the burden of antepartum and postpartum stillbirths, which can then further guide interventions. Using a mortality audit cycle of the circumstances surrounding deaths is an established quality improvement strategy that can highlight breakdowns in clinical care or processes at the local district level and ultimately reduce the missed opportunities to prevent such deaths [32]. We recommend that INAP uses this for stillbirth deaths and works towards creating an enabling environment that supports such an assessment with the health system. Furthermore, it would be prudent for the INAP to consider available recommendations to improve data availability and quality in health facilities from the antenatal care period to delivery to make reasonable progress in the reduction of stillbirths [2,4,11,55].

In conclusion, this study provides descriptive epidemiology on a large number of stillbirths from the state of Bihar, highlighting the unacceptably high burden of stillbirths and that urgent attention is needed to address the missed opportunities in the health system to reduce this burden. With INAP in place, the findings of this study are timely and pertinent to further strengthen the INAP approach to improve the quality and quantity of stillbirth data to avoid this needless loss of lives. It is important that India should aspire to register and document stillbirths in a systematic and standardised manner to bridge the knowledge gap for appropriate actions to reduce stillbirths.

## Supporting information

S1 STROBE Checklist STROBE checklist for the study. (DOC)

S1 COREQ Checklist COREQ checklist for the study. (DOCX)

S1 Data Data file for the study. (XLS)

## Abbreviations

ASHA: accredited social health activist
CI: confidence interval
DHS: Demographic and Health Surveys
INAP: India Newborn Action Plan
VDRL: Venereal Disease Research Laboratory
