# Supplementary material for: Identification of factors associated with stillbirth in the Indian state of Bihar using verbal autopsy: A population-based study
Source: PLoS Med. 2017 Aug 1;14(8):e1002363. doi: 10.1371/journal.pmed.1002363 (PMC5538635; doi:10.1371/journal.pmed.1002363)
Supplement: S1 COREQ Checklist — (DOCX) [file pmed.1002363.s002.docx]

Consolidated criteria for reporting qualitative studies (COREQ): 32-item checklist

| **No** | **Item** | **Guide questions/description** |  |
| --- | --- | --- | --- |
| **Domain 1: Research team and reflexivity** | | | |
| Personal Characteristics |  |  |  |
| 1. | Interviewer/  facilitator | Which author/s conducted the interview or focus group? | The interviews were conducted by interviewers trained in the study procedures. |
| 2. | Credentials | What were the researcher's credentials? *E.g. PhD, MD* | The researcher credentials include PhD, MD, MSc, and BA. |
| 3. | Occupation | What was their occupation at the time of the study? | Researcher’s occupations at the time of the study include social scientist, research fellow, research assistant, study coordinator, PI and research professor. |
| 4. | Gender | Was the researcher male or female? | Two of the 7 researchers are women. |
| 5. | Experience and training | What experience or training did the researcher have? | Research fellow/assistants had a minimum of 3 years´ experience in social sciences and/or qualitative research; study coordinator had 12 years of experience; social scientist had 15 years; and the research professors of over 20 years of experience in global health and quantitative/qualitative population research. |
| Relationship with participants |  |  |  |
| 6. | Relationship established | Was a relationship established prior to study commencement? | The interviewers did not have an ongoing relationship with the participants. |
| 7. | Participant knowledge of the interviewer | What did the participants know about the researcher? e*.g. personal goals, reasons for doing the research* | The participants were informed the name of the interviewer, the organisation s/he represented for the study, the purpose of the study, interview details including time needed and content, benefit of this information for population at large, contact details of the primary investigator and of the Ethics Committee that provided approval for the study as part of the formal informed consent process. |
| 8. | Interviewer characteristics | What characteristics were reported about the interviewer/facilitator? e.g. *Bias, assumptions, reasons and interests in the research topic* | Data were collected by trained interviewers. |
| **Domain 2: study design** | | | |
| Theoretical framework |  |  |  |
| 9. | Methodological orientation and Theory | What methodological orientation was stated to underpin the study? *e.g. grounded theory, discourse analysis, ethnography, phenomenology, content analysis* | Content and thematic analysis. |
| Participant selection |  |  |  |
| 10. | Sampling | How were participants selected? *e.g. purposive, convenience, consecutive, snowball* | Multi-stage sampling to arrive at a representative sample of participants. |
| 11. | Method of approach | How were participants approached? e*.g. face-to-face, telephone, mail, email* | Participants were approached face-face. |
| 12. | Sample size | How many participants were in the study? | 125,920 households. |
| 13. | Non-participation | How many people refused to participate or dropped out? Reasons? | A total of 16, 231 households (12.9%) did not participate in this study. Among these 15,167 (12.1%) were not available for interview post 3 contacts, 782 (0.6%) had migrated post enumeration, and 282 (0.2%) had other varied reasons for non-participation. |
| Setting |  |  |  |
| 14. | Setting of data collection | Where was the data collected? e*.g. home, clinic, workplace* | Data were collected at home of the participants. |
| 15. | Presence of non-participants | Was anyone else present besides the participants and researchers? | Verbal autopsy interviews benefit from presence of key informants. In this case, this included people with knowledge of context during labour and delivery of the child who was stillborn. Some interviews were done in the presence of key informants, as indicated by the study participant. |
| 16. | Description of sample | What are the important characteristics of the sample? *e.g. demographic data, date* | Majority of the interviews were provided by the mothers of the stillborn children, with a small number given by other relative. |
| Data collection |  |  |  |
| 17. | Interview guide | Were questions, prompts, guides provided by the authors? Was it pilot tested? | Yes and yes. |
| 18. | Repeat interviews | Were repeat interviews carried out? If yes, how many? | None. |
| 19. | Audio/visual recording | Did the research use audio or visual recording to collect the data? | No. |
| 20. | Field notes | Were field notes made during and/or after the interview or focus group? | Field notes were made when collecting the data. |
| 21. | Duration | What was the duration of the interviews or focus group? | Duration of the interviews were variable from 20-40 minutes. |
| 22. | Data saturation | Was data saturation discussed? | Data saturation was discussed. |
| 23. | Transcripts returned | Were transcripts returned to participants for comment and/or correction? | The complete verbatim was read to the participant by the interviewer after completing it. Any corrections that were made were made only at that time. |
| **Domain 3: analysis and findings** | | | |
| Data analysis |  |  |  |
| 24. | Number of data coders | How many data coders coded the data? | NA. Framework analysis was used. |
| 25. | Description of the coding tree | Did authors provide a description of the coding tree? | NA. |
| 26. | Derivation of themes | Were themes identified in advance or derived from the data? | Themes identified were derived from the data. |
| 27. | Software | What software, if applicable, was used to manage the data? | Ms Excel. |
| 28. | Participant checking | Did participants provide feedback on the findings? | Participants did not provide feedback on the findings. |
| Reporting |  |  |  |
| 29. | Quotations presented | Were participant quotations presented to illustrate the themes / findings? Was each quotation identified? e*.g. participant number* | Yes, identified as case number. |
| 30. | Data and findings consistent | Was there consistency between the data presented and the findings? | There was consistency between the data presented and the findings. |
| 31. | Clarity of major themes | Were major themes clearly presented in the findings? | The major themes were clearly presented in the findings. |
| 32. | Clarity of minor themes | Is there a description of diverse cases or discussion of minor themes? | There is no description of diverse cases or discussion of minor themes. |
